# Monogenic Inborn Errors of Immunity with impaired IgG response to polysaccharide antigens but normal IgG levels and normal IgG response to protein antigens

**DOI:** 10.3389/fped.2024.1386959

**Published:** 2024-06-12

**Authors:** Maria Fasshauer, Sarah Dinges, Olga Staudacher, Mirjam Völler, Anna Stittrich, Horst von Bernuth, Volker Wahn, Renate Krüger

**Affiliations:** ^1^ Immuno Deficiency Center Leipzig, Jeffrey Modell Diagnostic and Research Center for Primary Immunodeficiency Diseases, Hospital St. Georg, Leipzig, Germany; ^2^ Department of Pediatric Respiratory Medicine, Immunology and Critical Care Medicine, Charité - Universitätsmedizin Berlin, Corporate Member of Freie Universität Berlin, Humboldt-Universität zu Berlin, Berlin Institute of Health (BIH), Berlin, Germany; ^3^ Department of Human Genetics, Labor Berlin - Charité Vivantes GmbH, Berlin, Germany; ^4^ Department of Immunology, Labor Berlin - Charité VivantesGmbH, Berlin, Germany; ^5^ Berlin Institute of Health (BIH), Charité - Universitätsmedizin Berlin, Berlin, Germany; ^6^ Charité - Universitätsmedizin Berlin, Corporate Member of Freie Universität Berlin, Humboldt-Universität zu Berlin, Berlin Institute of Health (BIH), Berlin-Brandenburg Center for Regenerative Therapies (BCRT), Berlin, Germany

**Keywords:** IEI, primary immunodeficiency, polysaccharide, vaccines, pneumococcal infections, vaccination

## Abstract

In patients with severe and recurrent infections, minimal diagnostic workup to test for Inborn Errors of Immunity (IEI) includes a full blood count, IgG, IgA and IgM. Vaccine antibodies against tetanus toxoid are also frequently measured, whereas testing for anti-polysaccharide IgG antibodies and IgG subclasses is not routinely performed by primary care physicians. This basic approach may cause a significant delay in diagnosing monogenic IEI that can present with an impaired IgG response to polysaccharide antigens with or without IgG subclass deficiency at an early stage. Our article reviews genetically defined IEI, that may initially present with an impaired IgG response to polysaccharide antigens, but normal or only slightly decreased IgG levels and normal responses to protein or conjugate vaccine antigens. We summarize clinical, genetic, and immunological findings characteristic for these IEI. This review may help clinicians to identify patients that require extended immunologic and genetic evaluations despite unremarkable basic immunologic findings. We recommend the inclusion of anti-polysaccharide IgG antibodies as part of the initial routine work-up for possible IEI.

## Introduction

Inborn Errors of Immunity (IEI), formerly termed primary immunodeficiency diseases (PID), are a heterogeneous group of mainly monogenic diseases characterized by infections and/or immune dysregulation. The absence or impaired function of any immunologic component can determine the clinical presentation of an IEI. The heterogeneity of these diseases makes it difficult to direct routine laboratory work up (1, 2). When IEI with antibody deficiency are suspected (e.g., in patients with recurrent infections of the airways, the ears, the meninges, or the skin), the basic immunologic evaluation recommended by the German AWMF guideline (dating back to 2011), comprises a full blood count and measurement of IgG, IgA, and IgM levels (3). Once B-cell defects are suspected and serum immunoglobulins are detectable, evaluation of the IgG response to protein as well as polysaccharide antigens is recommended (4). To date, most primary care physicians do not routinely determine anti-polysaccharide (e.g., anti-pneumococcal) IgG/IgG2 antibodies or IgG subclasses when an IEI with antibody deficiency is suspected. A growing number of monogenic IEI has been reported, which may initially present with an impaired IgG response to polysaccharide antigens, whereas total IgG levels and response to protein (e.g., anti-tetanus toxoid IgG antibodies) and polysaccharide conjugate vaccines may be normal. Since polysaccharide-specific IgG antibodies fall within the IgG2 subclass fraction, IgG2 levels may also be decreased in these patients (5–7). Without the determination of anti-polysaccharide IgG antibodies and IgG subclasses, the diagnosis of several rare IEI may be missed or delayed. Delayed therapeutic interventions may then result in further infections and irreversible organ damage (e.g., bronchiectasis).

Patients with an impaired IgG response to polysaccharide antigens often suffer from recurrent or severe sinopulmonary infections with encapsulated bacteria that express abundant polysaccharide antigens on their surfaces e.g., *Streptococcus (S.) pneumoniae, Haemophilus (H.) influenzae* serotype b*,* and *Neisseria (N.) meningitidis* (6, 8). Severe invasive infections like meningitis, septicemia, or osteoarticular infections can also occur (7, 9, 10). An impaired IgG response to polysaccharide antigens can be diagnosed if wild-type infections with *S. pneumoniae* or vaccination with a pure polysaccharide vaccine (e.g., Pneumovax^™^) do not result in a significant increase of pneumococcal-specific IgG antibodies (11–13). Commercially available tests to assess IgM and IgA pneumococcal polysaccharide specific antibodies before and after pneumococcal polysaccharide based vaccinations are not routinely used but may be helpful in testing patients with IEI under IgG replacement therapy (14–16).

## Humoral immune responses to proteins and polysaccharides

**Figure 1:**
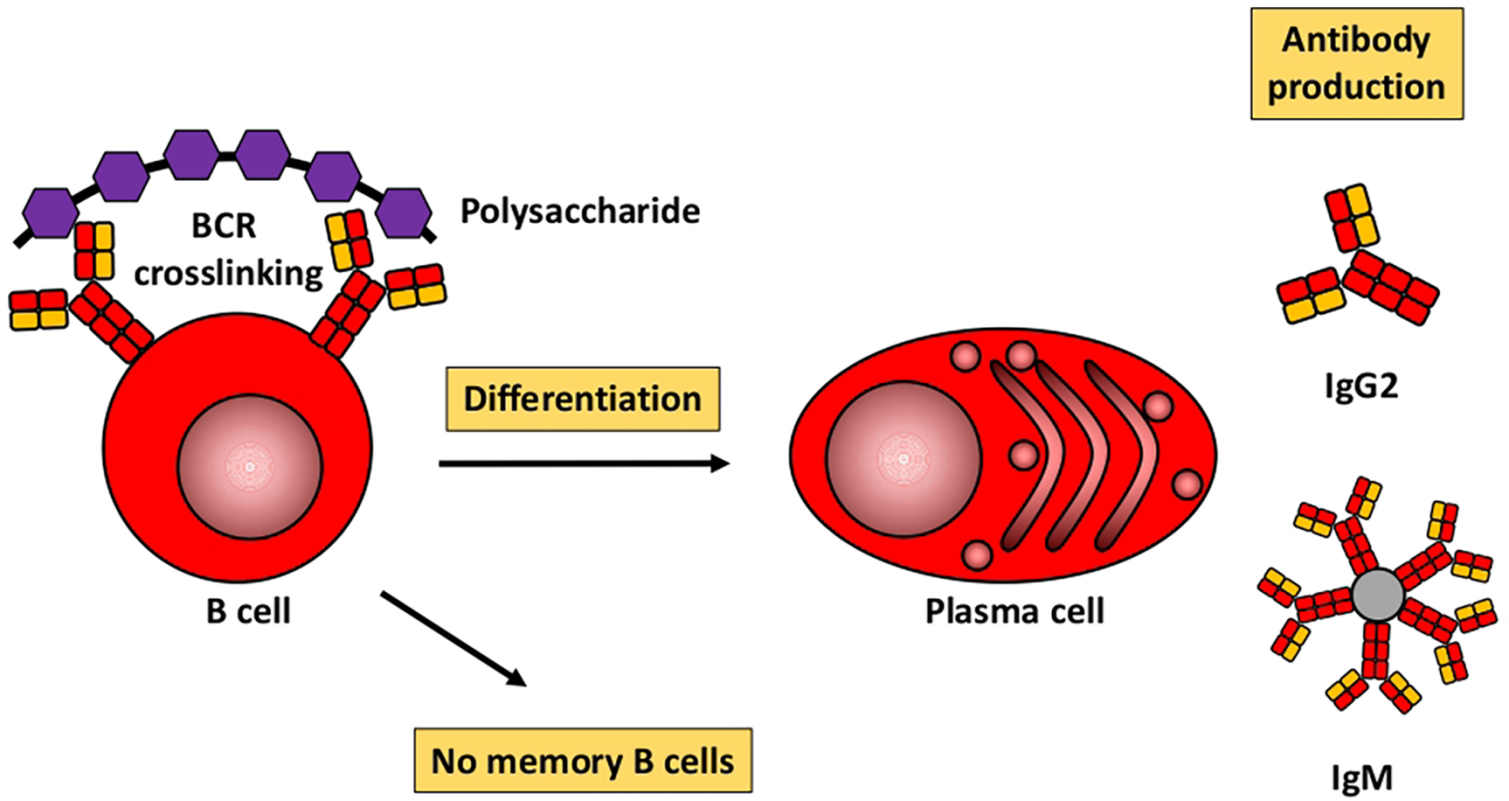
Humoral immune response to polysaccharides. Modified from Pollard et al. (17).

**Figure 2:**
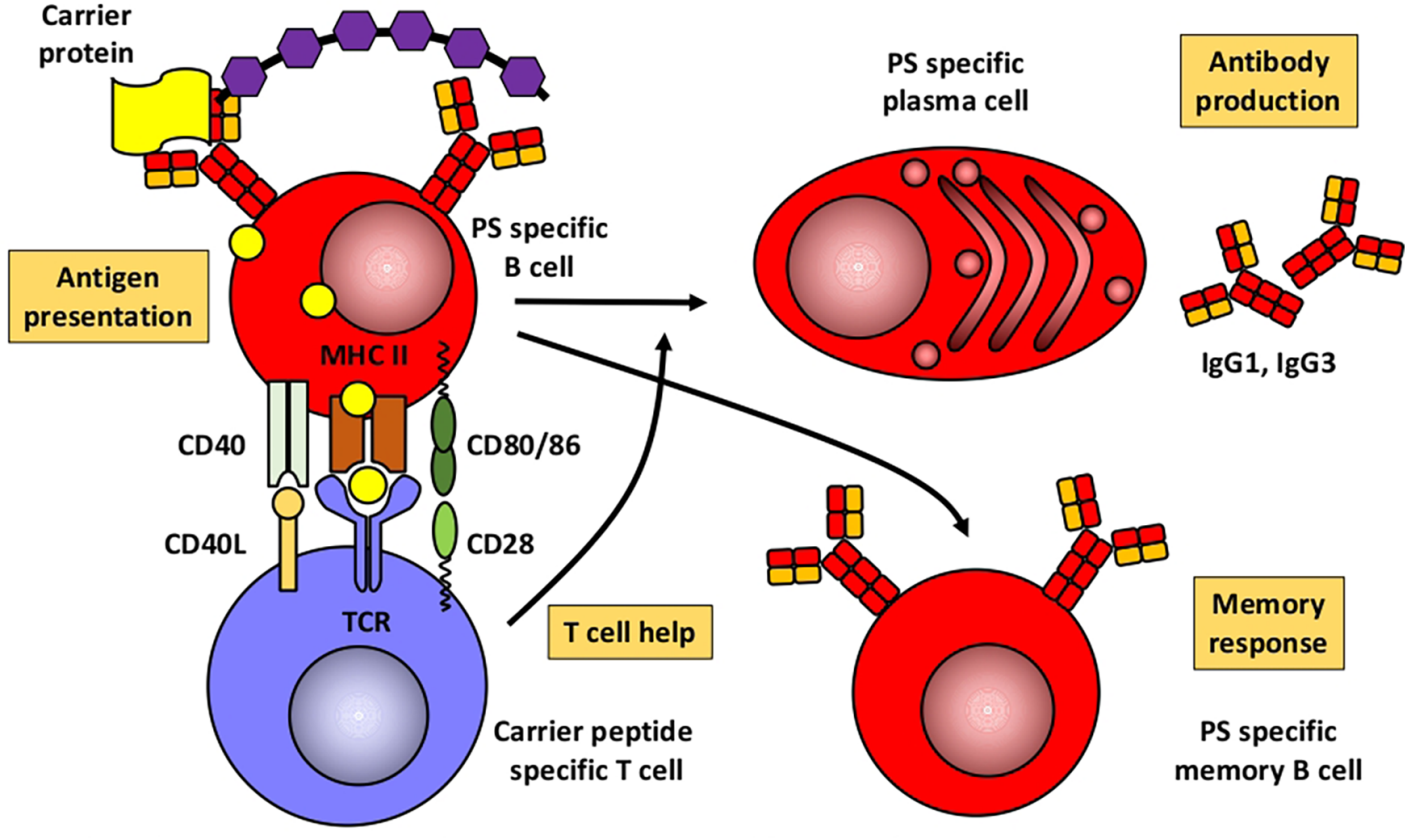
Humoral immune response to conjugated polysaccharides. Modified from Pollard et al. (17). PS, polysaccharide.

Based mainly on studies in mice, Figures 1, 2 illustrate the differences between immune responses against proteins and polysaccharides. Knowledge of these mechanisms is exploited for routine vaccination (17). The main immunological differences between the two mechanisms are summarized in Table 1. It explains the impaired IgG response to polysaccharide antigens described in some patients after polysaccharide vaccinations, usually with a 23-valent pneumococcal polysaccharide vaccine. In natural infections, for instance with pneumococci, protein and polysaccharide responses occur in parallel, in contrast to vaccine responses. The review by Gingerich and Mousa summarizes the structure of the bacteria and the targets of antibody formation (18).

**Table 1:** From Pollard et al. (17).

Polysaccharides	Polysaccharide conjugates
Induction by BCR crosslinking	T cells required
T cells not required	Carrier protein presented by MHC II
B cells short lived	Long-term immunity
No memory induced	Memory established
B cell memory pool depleted	New memory B cells
Hyporesponsiveness?	Boosted upon reexposure
Predominantly IgM and IgG2	Predominantly IgG1 and IgG3
Low immunogenicity in young children	Immunogenicity in young children

BCR, B cell receptor; MHC, major histocompatibility complex.

Recent literature has focused on the question as to which polysaccharides response is “normal”, the missing standardization of measuring anti-polysaccharide antibodies, the differences in assessment specifically in conjugate-primed and non-primed individuals, excluding other underlying diseases, or the need to verify patient-reported respiratory tract infections in patients with an impaired response to polysaccharide antigens. This shows the importance of measuring anti-polysaccharide antibodies and evaluating an immune response to polysaccharide antigens, as it further enlightens immune response to certain microbes (19, 20). Over the last decade, next-generation sequencing (NGS) for detecting genetic variants in IEI has identified an ever-increasing number of genetic causes and led to more specific diagnoses (21). Some of the monogenic IEI lack anti-polysaccharide antibodies due to an impaired immune response to polysaccharide antigens. However, IgG in these IEI can be normal or slightly decreased and the response to protein (e.g., tetanus toxoid) or conjugate (e.g., polysaccharide-protein) vaccines can also be unimpaired. These IEI -presented below- mostly show other immunologic abnormalities in addition to an impaired IgG response to polysaccharide antigens. Our review is therefore not restricted to IEI with specific antibody deficiency (SAD), since SAD is strictly defined as an IEI with an impaired response to polysaccharide antigens as the only abnormal immunologic finding (13, 20, 22). Following these basic considerations, we will now discuss specific aspects of respective monogenic IEI. Common clinical, genetic, and immunologic findings are also summarized in Table 2.

**Table 2:** Monogenic IEI with impaired IgG response to polysaccharide antigens but normal IgG levels: Common clinical, genetic, and immunologic findings.

IEI**^a^**	Gene/chromosome	Inheritance pattern	Incidence/prevalence	Age at presentation	Clinical phenotype	Infections	Common pathogens	Immunological phenotype**^b^**
BTK/XLA	BTK; -Xq22.1	XL	1:200,000 –700,000	6–18 months	Infections when maternal IgG wanes, absent/small tonsils, chronic lung disease, Crohn's like disease, arthritis, slight increased risk of malignancies	Respiratory, ENT, skin, CNS, osteoarticular, intestinal	*S. pneumoniae*, *H. influenzae*, *S. aureus*, *Helicobacter* spp. (skin), *Pseudomonas* spp., Enteroviruses (CNS), *Giardia lamblia*, *Pneumocystis jirovecii* (rarely)	Low/absent IgG, IgM, IgA, IgE, low/absent B cells, neutropenia (prior to IgRT)
Wiskott-Aldrich syndrome	*WAS;* Xp11.23	XL	1:50,000–250,000	Early childhood	Bleeding/bloody diarrhea, eczema, vasculitis, inflammatory bowel disease, IgA nephropathy, malignancies	Respiratory, ENT, meningitis, sepsis	*S. pneumoniae, H. influenzae, Neisseria meningitis* spp., HSV, *Molluscum contagiosum, Candida albicans, Pneumocystis jirovecii,*	Thrombocytopenia, low platelet volume, high IgA and IgE with normal IgG and/or decreased IgM, low isohemagglutinin levels, lymphopenia with low CD8+ T cells, increased proportion of effector memory T lymphocytes, normal T-cell proliferation upon PHA but reduced T-cell proliferation upon anti-CD3 stimulation, autoimmune cytopenias
NEMO deficiency	*IKBKG;* Xq28	XL	1:250,000	<1 year	Very variable, thickened skin, eczematous rashes, conical teeth, absent sweat glands, thin, sparse hair (often with unusual twirling), frontal bossing, failure to thrive, chronic diarrhea, autoimmune phenomena (AIHA, arthritis, inflammatory bowel disease-like colitis)	Respiratory, ENT, gastrointestinal, skin, soft tissues and bones, CNS	*S. pneumoniae, S. aureus, H. influenzae, P. aeruginosa,* Mycobacteria/BCG, CMV, HSV, ADV, HPV, *Pneumocystis jirovecii*	low IgG, low IgG2, low IL-10 response upon TNF-stimulation, NK cell abnormalities
pDGS	*TBX1*; 22q11.2	Mostly *de novo*, AD	1:4,000	Neonatal	Cardiac anomalies, hypoparathyreoidism, facial, palatal or ear nose throat dysmorphism, gastrointestinal anomalies, autoimmunity, allergy	Respiratory, ENT	*S. pneumoniae, S. aureus, H. influenzae, M. catarrhalis, P. aeruginosa, Respiratory viruses*	Low CD3+ T cells, low TRECs and naïve CD4 and CD8, low IgG, IgA, IgM, low switched and non-switched memory B cells
MECP2-Duplication	*MECP2*; Xq28	XL	1:150,000 (Australia)	Neonatal, infancy	Muscle hypotonia, mental retardation, impaired speech development, epilepsy, recurrent, severe pneumonia	Respiratory, sepsis	*S. pneumoniae, H. influenzae*	Low IgG2/IgG4, very high CrP during infections
NFKB1 deficiency	*NFKB1;* 4q24	AD, rarely AR	*1:10,000*	School age to adulthood	Bronchiectasis, interstitial lung disease gastrointestinal inflammation, chronic diarrhea, liver cirrhosis, hepatic hemangioma and hepatitis, skin disease, arthritis, thyroiditis, vasculitis, diabetes, Addison disease, lymphoproliferation with splenomegaly, lymphadenopathy and hepatomegaly, lymphomas and solid-organ cancer	Respiratory, ENT, gastrointestinal, skin	*H. influenzae, Streptococcus* spp. *M. catarrhalis, Pseudomonas* spp., *C. difficile, Salmonella* spp. *Campylobacter jejuni, E. coli, E. faecalis, S. epidermidis,* Influenza virus, RSV, rhinovirus, norovirus, rotavirus, adenovirus, EBV, CMV, John Cunningham virus *C. albicans, Aspergillus sp., P. jirovecii*	Low B cells, reduced switched memory B cells, expansion of CD21low B cells, low NK cells, low IgA, IgG and/or IgM, autoimmune cytopenias
NFKB2 deficiency	*NFKB2;* 10q24.32	AD	>55 patients described	School age to adulthood	Aspetic meningitis, opticus neuritis, lymphoproliferation, malignancy, asthma, ACTH deficiency, growth hormone deficiency, hypothyreoidism, prolactemia, trachyonychia, alopecia, eczema gastrointestinal disease, arthritis, lymphocytic organ infiltration (CNS, lung)	Respiratory, ENT, skin, eyes, CNS	*Salmonella* spp., EBV, CMV, *Toxoplasma gondii, Giardia lamblia*, *Candida* spp., *P. jirovecii*	Low marginal zone and switched memory B cells, total B cells can be normal, low IgG, IgA, IgM, autoantibodies (ANA, thyroperoxidase, anti-tyrosin-phosphatase, anti-Glutamat-Decarboxylase, anti-cytokine autoantibodies), autoimmune cytopenias
APDS	*PIK3CD;* 1p36.22 *PI3KR1;* 5q13.1	AD	<400 patients described	1–2 years	Lymphoproliferation, autoimmunity, malignancies, Infections, bronchiectasis	Mainly respiratory	*S. pneumoniae, H. influenzae, P. aeruginosa, S. aureus,* EBV, CMV, VZV, HSV, HPV, *Candida albicans*	High IgM, normal IgG/IgA, B-cell lymphopenia, increased transitional B cells, reduced memory B cells, CD4 lymphopenia (naïve CD4+ T cells)
CTLA4 deficiency	*CTLA4;* 2q33.2	AD	<200 patients described	School age	Lymphoproliferation, autoimmunity, malignancies, infections	Respiratory, gastrointestinal	*H. influenzae, S. pneumoniae, Salmonella enteridis, S. aureus,* EBV, CMV, HSV	Low IgG, T-cell lymphopenia (especially CD4+ T cells), B-cell lymphopenia (decreased B cell maturation), NK-lymphopenia
Ataxia–telangiectasia (A-T)	*ATM*; 11q22.3	AR	<1–9: 100,000	Early childhood	Progressive cerebellar ataxia, oculocutaneous telangiectasia, high incidence of malignancy (leukemia/lymphoma), endocrine abnormalities, immune dysregulation, autoimmunity, granulomas	Respiratory, invasive infections, warts, granulomas due to vaccine-strain rubella virus	*H. influenzae, H. parainfluenza, S. aureus, S. pneumoniae, E. coli, S. viridians, P. aeruginosa, C. albicans,* RSV, VZV, EBV, HHV6, Rubella vaccine virus	Increased AFP level, defects in T cell receptor (TCR) and B cell receptor (BCR) rearrangement causing (T and/or B cell) lymphopenia; (specific) antibody deficiency, 10%–20% hyper-IgM phenotype
Nijmegen breakage syndrome (NBS)	*NBS1;* 8q21-24	AR	1:100,000	Neonatal	Syndromic features: progressive microcephaly, prominent midface, sloping forehead, retrognathia, prominent nasal bridge and nose, large ears, café au lait spots, clino- and/or syndactyly, developmental delay, increased risk of malignancies (lymphomas and solid tumors), recurrent sinopulmonary infections females: primary ovarian failure	Respiratory, skin	*S. pneumoniae, H. influenzae*, Mycobacteria, recurrent HSV, severe or chronic EBV, CMV, HBV, HCV recurrent VZV, Rubella vaccine virus, *C. albicans*	(Combined) immunodeficiency—variable
DNA Ligase-4 syndrome	*LIG4*; 13q33.3	AR	>50 patients described	Neonatal	Microcephaly, facial features (resembling NBS), short stature, developmental delay, malignancy (lymphoma/ leukemia)	Respiratory, ENT, diarrhea	*S. pneumoniae*, *H. influenzae*, *Salmonella* spp., *Acinetobacter* spp., *C. albicans*, EBV, CMV, *Parainfluenza virus*	(Combined) Immunodeficiency, pancytopenia, myelodysplastic syndrome
Hyper IgE Syndrome (*STAT3*)	*STAT3;* 17q21.2	AD	1:11,000–1:100, 000	Neonatal, infancy	Severe eczema, bone brittleness (fractures), retention of decidual and delayed dentition of permanent teeth, scoliosis, coarse face, newborns: “prolonged exanthema toxicum infantum”, adults: aortic aneurysm, increased risk for lymphoma	Skin infections/abscesses, otitis, pneumonia	*S. aureus S. pneumoniae C. albicans*	IgE elevated, Eosinophils elevated, low IgG2, low memory B cells, low follicular helper cells

ACTH, adrenocorticotropic hormone; AD, autosomal dominant; AFP, Alpha Feto Protein; AIHA, autoimmune hemolytic anemia; ANA, antinuclear antibodies; AR, autosomal recessive; BCG, Bacille Calmette-Guérin, C. albicans, Candida albicans; CMV, cytomegaly virus; CNS, central nervous system; EBV, epstein barr virus; ENT, ear nose and throat; E. coli, Escherichia coli; E. faecalis, Enterococcus faecalis; ENT, ear nose and throat; HBV, Hepatitis B virus; HCV, Hepatitis B virus; HHV6, human herpes virus 6; HSV, Herpes simplex virus; H. influenzae, Haemophilus influenzae; NBS, Nijmegen breakage syndrome; RSV, respiratory syncytial virus; S. aureus, Staphylococcus aureus; sp., species; S. pneumoniae, Streptococcus pneumoniae; S. viridans, Streptococcus viridans; VZV, Varizella zoster virus; XL, X-linked.

^a^ For references see text.

^b^ In addition to an impaired IgG response to polysaccharide antigens.

## Hypomorphic mutations in *BTK*

X-linked agammaglobulinemia (XLA; M. Bruton) was first described in 1952 (23). It is an IEI with an estimated incidence of 1:200,000–700,000 live births. More than 600 pathogenic variants within *BTK* (encoding Bruton's Tyrosine Kinase) on chromosome Xq22.1 have been described to date (24). XLA patients have low or absent peripheral B cells and most patients have absent or very low IgG, IgA and IgM. Prior to IgG replacement therapy (IgRT) neutropenia is frequently observed (25–27). Without IgRT patients present with recurrent bacterial and viral infections beyond 3–6 months of age, once maternal IgG levels wane. Otitis media and pneumonia are the most frequent infections, severe invasive infections (meningitis, septic arthritis) as well as skin and gastrointestinal infections can also occur. Airway and invasive infections are mostly caused by *S. pneumoniae, H. influenzae* and *S. aureus. Pseudomonas* and *Helicobacter species* have been reported in chronic skin infections (28, 29). XLA patients are also susceptible to enteroviruses (e.g., poliovirus, coxsackievirus, echovirus) (30), that should be suspected in XLA patients with meningoencephalitis. *Giardia lamblia* can cause chronic diarrhea. In older children and adult XLA patients, chronic lung disease and autoimmunity (arthritis, enteropathy) is frequently reported, despite adequate IgG levels under IgRT (31, 32). Case studies have described patients with hypomorphic mutations in *BTK* who have no or only slightly decreased IgG levels, respond well to tetanus-toxoid vaccinations, but cannot mount an IgG response to polysaccharide antigens (33, 34).

## Wiskott-Aldrich syndrome

The X-linked Wiskott-Aldrich syndrome (WAS) was first described in 1937 (35), and further delineated by R. Aldrich in 1954 (36). Positional cloning in the 1990s identified mutations in the gene encoding the Wiskott-Aldrich syndrome protein (WASp) as the cause of the disease (37). WASp is a crucial regulator of various functions in hematopoietic and immune cells, including cytoskeletal reorganization, immune synapse formation, and intracellular signaling (38, 39). Genotype-phenotype correlations for more than 150 mutations are sparse. WAS has an estimated incidence of about 4 per million live male births and presents a wide spectrum of clinical phenotypes (40). The disease commonly manifests as a bleeding disorder coupled with increased susceptibility to infections (encapsulated pathogens, viruses, opportunistic pathogens), autoimmunity (e.g., eczema, autoimmune cytopenias, vasculitis, inflammatory bowel disease, IgA nephropathy), and an increased risk of hematological malignancies (41). Typically, both cellular and humoral immunity are affected. Common laboratory findings include thrombocytopenia with reduced platelet volume and lymphopenia, often due to T-cell loss, while B-cell counts usually remain stable. Platelet volume may normalize in patients post splenectomy, so normal platelet volume does not exclude WAS in patient post splenectomy (42). Decreased CD8+ T cell count and function, reduced natural killer (NK) cell cytotoxicity, and impaired regulatory T cell (Treg) cell function contribute to the immunodeficiency and autoimmunity (43). WASp gene mutations disrupt actin polymerization in WAS-deficient T cells, affecting T- and B-cell interactions that are crucial for memory cell development and isotype switching (44). As a result, immunoglobulin levels are often altered (typically elevated IgA and IgE, normal IgG, and decreased IgM), isohemagglutinin titers tend to be low, and the specific IgG antibody response to various antigens is often insufficient (e.g., pneumococcal polysaccharide and tetanus toxoid) (45).

## Nuclear factor-kappa B essential modulator (NEMO) deficiency

NEMO deficiency syndrome is a rare, combined immunodeficiency that was concurrently elucidated in three seminal publications in the early 2000s (46–48). It is caused by hypomorphic mutations in the X-linked *IKBKG* (also known as inhibitor of nuclear factor kappa-B kinase subunit gamma, NEMO) gene. Genetic analysis is complicated through homologous pseudogene sequences. NEMO is a key player in the NF-κB pathway, consequently, crucial for the development and function of the ectoderm, the immune system, and bones. The prevalence of NEMO deficiency is about 1:250,000. The phenotype spectrum in NEMO deficiency is exceedingly broad. 80% of patients exhibit ectodermal dysplasia (EDA) characterized by thickened skin, eczema, conical teeth, absent sweat glands, and hypotrichosis (49). Immune deficiency in NEMO patients renders them susceptible to recurrent respiratory tract infections, encapsulated bacteria, severe viral (CMV) and opportunistic infections (pneumocystis, environmental mycobacteria) (50, 51). Typically, patients present with severe pneumococcal infections despite vaccination, early episodes of meningitis, or deep tissue infections (51). Besides EDA and immune deficiency, a subset of patients also suffers from osteopetrosis, lymphedema, and/or autoimmunity. Characteristic laboratory findings are normal or low IgG (especially low IgG2) levels with elevated or decreased IgM or IgA levels. Immune response to stimulation with LPS, IL-1ß and TNF may be decreased, and IgG response to pneumococcal polysaccharide vaccination is in most cases insufficient. Class-switched B cells tend to be reduced, and/or NK cell activity abnormal (49–54).

## DiGeorge syndrome/22q11.2 deletion syndrome

Neonatal co-occurrence of thymic aplasia and hypoparathyroidism was initially described by DiGeorge in 1965 (55) and later called DiGeorge syndrome (DGS). The 22q11.2 microdeletion is both the most common cause of DGS and the most common human chromosomal microdeletion syndrome in general, with an incidence of 1:4000 live births. Over 90% of the cases are *de novo* mutations causing haploinsufficiency of *TBX1*, a transcription factor involved in patterning of the third pharyngeal pouch. The genotype-phenotype correlation is weak and there is great clinical variability. While less than 1% of DGS patients are athymic (complete DGS), the majority presents with less severe thymic hypofunction (partial DGS, pDGS). pDGS is defined as <1,500/mm^3^ CD3+ T cells and at least one of the following: (1) cardiac malformation, which is the main cause of death, (2) hypocalcemia, or (3) facial or palatal dysmorphism. Gastrointestinal anomalies, autoimmunity, mostly cytopenias and thyroid disease, and allergic manifestations are frequent. Patients present with recurrent infections including sinusitis, otitis media, bronchitis, and pneumonia (56). T-cell lymphopenia of varying severity is the most common feature, though 20% of patients have normal T-cell counts (57). Over time, T cells meet levels of age-matched controls due to homeostatic expansion, resulting in decreased T-cell receptor repertoire (56, 58). Lymphoproliferation upon stimulation with mitogens is mostly adequate. Delayed B-cell maturation, reduced numbers of naïve and unswitched memory B cells and peripheral class switched memory B cells, as well as hypogammaglobulinemia and isolated IgA or IgM deficiency may occur (58). Impaired IgG response to polysaccharide antigens was reported in 40 to 55% of patients, most likely due to impaired assistance of thymus-derived T cells to the humoral immune response (56, 59).

## Methyl-CpG-Binding protein 2 (MECP2) duplication syndrome

*MECP2* duplication syndrome is an X-linked disorder first described in 2005 (60, 61). For a recent review, see (62). In Australia, incidence is estimated at 1: 150,000 live births, respective estimates from Europe are not available. More than 500 patients have been reported to date (reviewed in (62). Penetrance is 100%, affected males present with neonatal muscular hypotonia, severe intellectual disabilities, recurrent and frequent seizures, low sensitivity to pain, as well as severe infections of the lower airways. During infections, hyperinflammation with very high levels of inflammatory markers [e.g., C-reactive protein (CrP)] can be observed. The majority of affected patients die from severe pneumonia in childhood or as young adults. Most patients have low IgG2 and IgG4 subclasses and an impaired response to polysaccharide antigens (60, 63–71).

## Nuclear factor-kappa B1 (NFKB1) deficiency

NFKB deficiency is an IEI with immune dysregulation. *NFKB1* loss-of-function or hypomorphic variants, first reported in 2015 (72), are inherited autosomal dominant resulting in haploinsufficiency (73). Recently, in 2021, the first autosomal recessive variant leading to NFKB1 deficiency was identified (74). NFKB1 deficiency affects up to 1 in 10,000 individuals. Three quarters of the patients fulfill the criteria of CVID. NFKB1 deficiency accounts for 4 to 5% of genetically resolved CVID cases, thus representing the most common monogenetic etiology of CVID (73, 75). Incomplete penetrance and age-depended disease progression were reported (73). Patients present with respiratory, gastrointestinal, skin, and opportunistic infections, mostly bacterial. Lymphoproliferation and autoimmunity, particularly cytopenias, are characteristic. There is a high frequency of multiorgan autoinflammation, noninfectious enteropathy, hepatopathy, and malignancy. Hypogammaglobulinemia and low IgA and/or IgM are common findings. A relative increase of CD21^low^ B cells was associated with autoimmune and lymphoproliferative phenotypes. CD4 T-cell defects and a T_H_1 and proinflammatory cytokine predominance are possible. Decreased numbers of circulating NK cells are frequent (75). An impaired IgG response to polysaccharide antigens was described in 53%–65% of patients with NFKB1 deficiency. NF-κB regulates the expression of activation-induced cytidine deaminase, an enzyme mediating class switch recombination, thereby possibly impairing humoral immunity if mutated (76, 77).

## NFKB2 deficiency

In 2013, the first patients with *NFKB2* mutations were reported with a phenotype of CVID and adrenocorticotropic hormone (ACTH) insufficiency, a syndrome called DAVID (deficient anterior pituitary with CVID) (78). *NFKB2* encodes the precursor p100 and is located at 10q24.32. The reported damaging mutations follow an autosomal dominant pattern. To date, 27 cases of DAVID syndrome and a total of at least 50 cases of CVID with and without DAVID syndrome with confirmed pathological variants in the *NFKB2* gene have been published (79). Clinical expressivity and penetrance of *NFKB2*-related diseases are heterogeneous. Of note, inflammatory, autoimmune, and malignant manifestations often go beyond the common spectrum seen in CVID (79). A key feature, often preceding other symptoms, is autoimmune alopecia. Patients may also present initially with respiratory infections, diarrhea, and arthritis. Over time, about 40% of mutation carriers develop ACTH deficiency, sometimes accompanied by growth hormone and thyroid stimulating hormone deficiencies (80). Recurrent and severe infections with Herpesviridae, candida, and opportunists such as *Pneumocysits (P.) jivorecii* were reported. Most cases present with hypogammaglobulinemia and reduced marginal zone and switched memory B cells. Total B-cell, T-cell and NK-cell counts are often unimpaired. An impaired IgG response to polysaccharide antigens is present in almost half of the patients. The NFKB2 pathway is indispensable for both B-cell maturation, survival and function and T follicular helper cell generation, explaining humoral immunodeficiency (79).

## Activated phosphoinositide 3-kinase δ syndrome (APDS)

APDS is caused by either pathogenic gain-of-function variants in *PIK3CD* (APDS1) or loss-of-function variants in *PIK3R1* (APSD2) (81). First described in 2013, APDS results in hyperreactive mTOR signaling and subsequent immune dysregulation (82). Inheritance is typically autosomal dominant, with onset of symptoms in the second year of life. Patients suffer from sinopulmonary infections resulting in bronchiectasis, B-cell lymphoproliferation (non-malignant or malignant), and autoimmune/inflammatory conditions [e.g., autoimmune hemolytic anemia, enteropathy, and immune thrombocytopenia (ITP)]. They are at increased risk of lymphoma, hemophagocytic lymphohistiocytosis (HLH), enteropathy, and occasionally developmental delay. Typical pathogens causing bacterial infections include *S. pneumoniae, H. influenza, Pseudomonas (P.) aeruginosa and S. aureus.* Viral infections are commonly caused by Herpesviridae (EBV, CMV, VZV, HSV) or HPV, and fungal infections by *C. albicans*. The immunological phenotype in APDS is characterized by a diminished antibody response to polysaccharide antigen, elevated IgM, normal IgG and IgA, B-cell lymphopenia, increased transitional B cells, reduced memory B cells, and CD4 lymphopenia, especially naïve CD4+ T cells (83). The CD4/CD8 ratio is decreased (81, 84, 85).

## Cytotoxic T lymphocyte antigen 4 (CTLA4) deficiency

Heterozygous pathogenic variants in *CTLA4* were initially reported in 2014. They lead to CTLA4 deficiency through haploinsufficiency, inadequate dimerization, or insufficient effector binding (86). The dominant inheritance pattern is accompanied by incomplete penetrance. Since CTLA4 plays a vital role in Tregs function and suppression of T-cell response, its deficiency results in immune dysregulation. Symptom onset typically occurs around 11 years of age, with a highly variable presentation. The immunological phenotype of CTLA4 deficiency primarily exhibits hypogammaglobulinemia, with lack of anti-polysaccharide antibodies, accompanied by significant reductions in T-cell lymphopenia, particularly evident in CD4+ T cells. B-cell lymphopenia with impaired B-cell maturation, and reduced NK cells are also notable features. Clinical manifestations encompass lymphoproliferation, autoimmune conditions (predominantly cytopenia or gastrointestinal involvement), increased susceptibility to infections, particularly of the respiratory tract [viral (EBV, CMV, HSV), bacterial (*H. influenzae*, *S. pneumoniae*, *Salmonella enteritidis*, *S. aureus*) and fungal (Candida species, Aspergillus species)], as well as gastrointestinal and neurological features. Additionally, patients face an increased risk of malignancies such as lymphoma and gastric cancer, often associated with EBV (87).

## Ataxia–telangiectasia (A-T)

Ataxia–telangiectasia (A-T; Louis-Bar Syndrome) was first described in 1941 (88) and further characterized by Boder and Segwick 1958 (89). A-T is caused by mutations in the gene *ATM* (Ataxia Telangiectasia, Mutated) on human chromosome 11 (11q22.3) (90). The estimated incidence ranges from 1:40,000 to 1:300,000 live births with higher incidences in countries with high rates of consanguinity (91, 92). Several hundred pathogenic and likely pathogenic variants as well as >>1,000 variants of unknown significance (VUS) have been documented in the large gene comprising 60 exons, without any hotspots for mutations (see https://www.LOVD.nl/ATM). ATM protein has a crucial role in repair of DNA double strand breaks caused by radiation, oxidative or other genotoxic stress (90, 93). A-T is inherited in an autosomal recessive order. In non-consanguineous families, most A-T patients are compound heterozygote (94). Clinical features can vary but progressive ataxia noted once children start walking and oculocutaneous telangiectasia (in patients ages 5 years and older) are most prominent findings. Frequent sinopulmonary infections due to immunodeficiency, malignancies (in 1/4 of patients) as well as chronic lung and liver disease, gonadal insufficiency/sterility and premature aging are also reported. Life expectancy is limited mostly due to lung disease and malignancies [for review see (95, 96)]. Approximately 95% of individuals with A-T have marked elevated serum alpha-fetoprotein (AFP) levels (97). Some degree of immunodeficiency is present in approximately 2/3 of A-T patients, with lymphopenia, decreased levels of IgG, IgA, IgM, IgE and/or IgG subclasses and/or an impaired IgG response following vaccinations or infections being observed (98, 99). A-T patients may also may present with an hyper-IgM phenotype (100). A hyper-IgM phenotype or IgA deficiency indicate a poor prognosis (101). Of note, approximately 50% of A-T patients are detected by the newborn screening for severe combined immunodeficiency (SCID) based on low numbers of naïve T cells (102). Immunological impairment in most A-T patients sustains over time, thus frequent reevaluations of immunologic parameters are mostly not indicated (103). Immune dysregulation and autoimmunity in A-T such as immune thrombocytopenia (ITP), arthritis, and vitiligo is also observed [reviewed in (95)]. Chronic granuloma caused by live rubella vaccine virus have been described predominantly in patients with A-T and other DNA repair disorders [reviewed in (104, 105)].

## Nijmegen breakage syndrome (NBS)

NBS is a rare autosomal recessive disease that was first described by researchers at the University of Nijmegen (106, 107). NBS is caused by mutations in *NBN* (mapped to human chromosome 8q21.3) that encodes the protein Nibrin, also termed NBS1 or p95 (108, 109). Nibrin plays an important role in DNA damage response and DNA repair (110). The majority of patients affected by NBS are of Slavic origin due to a founder mutation which leads to protein truncation (109). Prevalence is estimated to approximate 1:100,000 live births (110, 111). The main features of NBS include microcephaly, usually since birth, typical facial appearance with prominent midface, sloping forehead, retrognathia, prominent nasal bridge and nose, large ears and mild growth retardation. Some patients may also have café au lait spots, clinodactyly and syndactyly. Psychomotor development may initially be normal or mildly impaired, but intellectual abilities often decline. Female NBS patients are characterized by primary ovarian failure. Patients may suffer from recurrent respiratory and urinary tract infections, gastroenterocolitis, autoimmune diseases, and have a pronounced predisposition to malignancy due to chromosomal instability and radiation hypersensitivity, with more than 40% of NBS patients developing a malignancy (mainly lymphomas) by the age of 20 years (111–113). Immunological abnormalities found in most patients but are highly variable: T-cell lymphopenia (CD4+ T cells and/or CD8+ T cells) and elevated number of NK cells have been reported. IgG and IgA deficiency can occur, an impaired IgG response to pneumococcal polysaccharide antigens is observed in the majority of patients (75%), often combined with decreased levels of IgG2 and IgG4 subclasses (114). In contrast to A-T, immunodeficiency in NBS patients may progress over time (111, 112). Despite confirmed immunodeficiency, some NBS-patients do not suffer from frequent infections and do not require prophylactic antibiotics or IgRT (111, 112, 114).

## DNA ligase 4 (LIG4) syndrome

DNA Ligase IV (LIG4) syndrome, also known as DNA Ligase IV deficiency or Ligase 4 syndrome, is a very rare autosomal recessive disorder that also belongs to the group of hereditary diseases associated with defects in cellular responses to DNA damage. The syndrome results from pathogenic variants in the gene encoding DNA ligase IV (*LIG4*) that was mapped to chromosome 13q33.3. LIG4 syndrome is caused by homozygous or, more often, compound heterozygous hypomorphic mutations in the *LIG4* gene. A genotype–phenotype correlation is suspected for single truncating mutations (115). The presentation of LIG4 syndrome is very heterogenous. Some individuals carrying a deleterious variant were reported to be asymptomatic (116). Common clinical findings are microcephaly, severe growth retardation, developmental delay and dysmorphic facial features, chronic liver disease and malignancy predisposition due to pronounced radiosensitivity (117, 118). Patients with hip dysplasia and other skeletal malformations were also reported. Immunologic findings comprise low IgG, variable immunodeficiency, and pancytopenia. Manifestation early in life has been reported for some patients, who presented with the above-mentioned clinical complex as well as severe combined immunodeficiency, radiosensitivity, chronic liver disease, and progressive bone marrow failure. Immunological findings include hypogammaglobulinemia and very low B cells with primarily IgG deficiencies causing sinopulmonary infections. Hypogammaglobulinemia can be accompanied by a decrease in IgA and IgM levels as well as T-cell abnormalities (117, 118). Patients with distal truncating pathogenic variants typically have a milder phenotype and may present later in life with hematological neoplasia with poor response to chemo- and radiotherapy (116, 117). Live expectancy is limited due to hematologic neoplasia, bone marrow failure, and chronic liver disease (117, 118).

## Autosomal dominant hyper IgE syndrome (HIES) caused by dominant negative mutations in *STAT3*

First described as “Job's Syndrome”, the term “Hyper-IgE Syndrome” (HIES) was later introduced because of high IgE-levels (119, 120). Several genetic variants associated with Hyper-IgE have been identified. Autosomal dominant HIES (AD-HIES) is characterized by (1) pulmonary infections, mainly due to *S. aureus* and *S. pneumoniae*, (2) bone brittleness and retention of decidual and delayed dentition of permanent teeth, (3) severe eczematous dermatitis and skin infections with *S. aureus* and *Candida* spp. (121). The identification of heterozygous, almost exclusively dominant-negative mutations in the gene that encodes the Signal transducer and activator of transcription 3 (*STAT3*), as genetic cause for AD-HIES explains the syndromic nature of HIES, affecting not only the immune system, but multiple further tissues (122). The reported prevalence among populations varies between 1 and 9: 100,000. (https://www.orpha.net/).

As in atopic dermatitis, the predominant immunological feature of AD-HIES is marked hypereosinophilia and elevated IgE (121, 123). It was shown early and repeatedly thereafter that IgG-responses against pneumococci and levels of IgG subclasses may be low, too (124–127). Thus, in patients with severe eczema normal levels of IgG subclasses and normal responses against pneumococci do not exclude AD-HIES, but low levels of IgG subclasses and/or impaired responses against polysaccharides are compatible with AD-HIES. Low IgG subclasses and/ or impaired humoral responses against pneumococci are probably caused by combined effects of less follicular helper T cells (producers of IL-21), impaired IL-6 signalling (and consequently low IL-21 production) and impaired responses to IL-21, that finally lead to less class-switched memory B cells in many patients with HIES caused by dominant negative mutations in *STAT3* (128–134). IgG2 subclass deficiency was described very early in AD-STAT3-HIES (125) and may contribute to the increased susceptibility to pneumococcal infections.

## Other rare IEI with an impaired IgG response against polysaccharide antigens

Other rare IEI may present with an impaired IgG response to polysaccharide antigens as a predominant finding (135), e.g., Syk (136) and MAGT1-deficiency (137); but larger case series or cohort studies are lacking.

## Discussion

Severe or recurrent infections with encapsulated bacteria (e.g., *S. pneumoniae, H. influenzae, N. meningitis species*) should prompt clinicians to evaluate patients for IEI, in particular when affected patients had been vaccinated against respective bacterial antigens. Apart from antibody deficiency, differential diagnostic considerations should include complement deficiency, asplenia or splenic impairment as in sickle cell disease, and defects in innate immunity such as Toll-like/IL1R defects e.g., IRAK4/MyD88 deficiency (12, 13). The majority of patients with recurrent or severe infections due to antibody deficiency (e.g., CVID, XLA) will be detected with the basic immunologic tests recommended by German AWMF guidelines (3). However, IgG, IgA, IgM, and even IgG responses to protein-based vaccines may (initially) be normal in patients with monogenic IEI with an impaired response to polysaccharide antigens. Thus, we would like to challenge current German guidelines because these IEI may be missed or the diagnosis delayed. An impaired response to polysaccharide antigens can be diagnosed once polysaccharide-specific antibodies do not increase following polysaccharide-based vaccinations or wild-type infections. In wild-type infections with *S. pneumoniae* protein as well as polysaccharide-specific antibodies to respective strains should be detectable in high/normal concentrations. Thus, low or absent pneumococcal specific IgG after microbiology-proven pneumococcal infection should prompt for further immunological and genetic workup. Commercially available tests for polysaccharide-specific total and IgG2 antibodies are mostly based on pneumococcal antigens. Patients with recurrent or severe bacterial infections and low specific pneumococcal antibodies should receive a “diagnostic” polysaccharide-based vaccination (e.g., Pneumomvax™) followed by measurement of the IgG vaccine response ≥4 weeks. Unfortunately, various serological and opsonophagocytic assays exist with limited comparability of the results. An international consensus for a standardized approach would be highly desirable. Determination of IgG subclasses may also be helpful, since polysaccharide-specific IgG antibodies are found within the IgG2 subclass fraction, thus IgG2 levels are often below age dependent normal ranges in patients with an impaired response to polysaccharide antigens (138). Of note, children below the age of 2 years respond poorly to polysaccharide antigens, therefore (diagnostic) vaccination with Pneumovax™ is not approved in children <2 years of age. Also, IgG2 and IgG4 levels are frequently low in infancy and are likely increase to normal levels within the first two years of live (138, 139). Diagnosing an impaired IgG response to polysaccharide antigens is therefore more challenging in children younger than 2 years than in older children and adults.

In this review, we listed respective IEI to be considered once an impaired IgG response to polysaccharide antigens has been diagnosed. The clinical characteristics shown in Table 2 imply that

(a) syndromic aspects as in DGS, *MECP2*-Duplication syndrome, A-T, NBS and Lig4-Deficiency,
(b) male gender (XLA, WAS, *MECP2* Duplication syndrome),
(c) a positive family history for infections or autoimmunity, and
(d) consanguinity

in the context of recurrent infections should alert physicians to perform immunologic (and genetic) testing beyond basic evaluations for IEI. In most listed IEI, phenotypic presentations can vary significantly within a pedigree, thus a selectively impaired response to polysaccharide antigens may be present in relatives from patients with more pronounced immunologic findings.

Genetic testing has become more available and less expensive over the last years. Therefore, Whole Exome or Whole Genome Sequencing (WES, WGS) has become a standard diagnostic procedure when IEI is suspected based on clinical and/or immunological findings in high income countries (140). We assume that a growing number of patients with an impaired IgG response to polysaccharide antigens or strictly defined specific antibody deficiency (SAD, see below) will show pathogenic variants in one of the listed genes. Family members of patients with respective IEI should be offered genetic testing to allow close follow-up and early therapeutic interventions when pathogenic variants are identified. Genetic diagnosis of IEI in young children also permits genetic counseling of parents with a desire to have more children.

Delayed diagnosis of IEI with antibody deficiency is common (141), often patients show permanent organ damage due to recurrent sinopulmonary infections when diagnosed: hearing impairment following recurrent otitis, sinus surgery in patients with chronic or recurrent rhinosinusitis, bronchiectasis/chronic lung disease due to recurrent pneumonia (142).

Early diagnosis and treatment of IEI with an impaired IgG response to polysaccharide antigens listed in this review cannot only prevent further infections and organ damage, but can also prevent specific short and long term complications and risks in specific IEI [i.e., bleeding in WAS, risk of malignancy due to exposure to ionizing radiation as well as risk of rubella-induced granuloma following live-rubella vaccination in IEI with DNA repair disorders (A-T, NBS, LIG4 deficiency)] (104, 143).

Treatment of patients with an impaired IgG response to polysaccharide antigens depends on clinical symptoms, however, patients with recurrent bacterial infections will benefit from antibiotic treatment/prophylaxis and/or IgRT. Of note, in patients with normal IgG levels and an impaired IgG response to polysaccharide antigens, IgG dosing and intervals should not be guided by through levels of total IgG but by (a) the clinical response and (b) pneumococcal specific antibodies and/or IgG2 levels (if decreased prior IgRT). In patients with a normal IgG response to protein-based vaccines (tetanus toxoid), non-live protein-based vaccinations are advisable irrespective of IgRT when protective antibody titers cannot be guaranteed in IgG preparations (in particular: Meningococci A, B, C, W, Y). The add-on effect of such vaccinations, however, has not been scientifically evaluated.

Inhalant antibiotics may be an option in individual patients with recurrent pneumonia and bronchiectasis despite IgRT and systemic antibiotic prophylaxis. It has been successfully applied in a patient with *MECP2*-Duplication syndrome (144) as well as in CVID patients (145). However, there are no controlled trials on inhalant antibiotics in pediatric IEI patients.

HSCT has become an option for a growing number of patients with IEI, the outcome is generally better when patients are transplanted before chronic infections occur, thus, patients with a clear indication for a curative HSCT (e.g., patients with WAS) will benefit from early diagnosis (146). HSCT has been successfully performed in selected cases with DNA repair disorders, mainly because of malignancies (147, 148). Early diagnosis of a DNA repair disorder prior to HSCT enables adjustment of conditioning regimen and minimization of radiation exposure.

More specific treatment options in order to prevent disease progression, organ damage and/or to improve quality of life that are available for distinct IEI mentioned in the article: Recent clinical observations show impressive improvement of dermatitis and almost complete control of skin infections in patients with HIES on Dupilumab [subcutaneously (s.c.)]—an antibody that blocks the IL4-receptor alpha chain (149–155). Abatacept (s.c.), a CTLA4-immunoglobulin fusion drug, has shown excellent treatment results with a good tolerability and safety profile in patients with CTLA4 and LRBA deficiency (156, 157). This has also been shown for the orally available Leniolisib, a small molecule selective inhibitor of PI3Kδ, in patients with APDS (81, 84, 85). In A-T, antisense oligonucleotide treatment has shown amelioration of neurological disease progression in patients with selected *ATM* mutations (158, 159). This therapy has to be initiated at an early disease state, underscoring the importance of timely diagnosis. Gene therapy may become an option for some of the listed IEI in the future (160, 161).

In this article, we purposely discussed patients that do not fulfill the strict criteria for SAD with a diminished antibody response to polysaccharide antigens following vaccination or wild-type infection as the only immunologic pathology (normal levels of IgG, IgA, IgM and IgG subclasses) (12, 13, 20, 22) for the following reasons: SAD is not a genetically distinct IEI. Indeed, genetic testing of patients with SAD may reveal mutations in various IEI genes described in this article. Also, IgG subclasses are not routinely tested in all countries, it is therefore unclear if patients with unknown IgG subclass levels do have strictly defined SAD. Furthermore, all patients with an impaired IgG response to polysaccharide antigens and frequent or severe bacterial infections will benefit from therapy, irrespective of additional immunologic abnormalities.

Lastly, this review underscores the importance of microbiology testing in patients with recurrent bacterial infections prior to antibiotic therapy. Once encapsulated bacteria are repeatedly detected, immunodeficiency with an impaired response to polysaccharide antigens may be suspected and diagnosed. Microbiology testing also helps to avoid non-effective antibiotic regimens due to resistances in patients that had multiple courses of antibiotics and select an effective prophylactic regimen if required.

Apart from monogenic IES listed in this manuscript, testing for polysaccharide specific antibodies is also helpful in patients with CVID and unclassified antibody deficiencies without an underlying genetic defect identified. Absent or low specific antibodies with no increase following a polysaccharide based vaccination should prompt clinicians to consider antibiotic prophylaxis or IgRT.

## Conclusions

In patients with suspected IEI due to severe or recurrent bacterial infections who have normal IgG levels and a normal IgG response to protein-based vaccines, testing for polysaccharide-specific antibodies and IgG subclasses can help to identify rare monogenic IEI early, initiate treatment earlier and, thus, prevent morbidity, mortality, and organ damage. In our opinion, new guidelines on diagnostic measures in IEI should include the determination of specific IgG antibodies and IgG subclasses.
